# Extreme learning machine: a new alternative for measuring heat collection rate and heat loss coefficient of water-in-glass evacuated tube solar water heaters

**DOI:** 10.1186/s40064-016-2242-1

**Published:** 2016-05-14

**Authors:** Zhijian Liu, Hao Li, Xindong Tang, Xinyu Zhang, Fan Lin, Kewei Cheng

**Affiliations:** Department of Power Engineering, School of Energy, Power and Mechanical Engineering, North China Electric Power University, Baoding, 071003 China; College of Chemistry, Sichuan University, Chengdu, 610064 China; College of Mathematics, Sichuan University, Chengdu, Sichuan, 610064 China; National Center for Quality Supervision and Testing of Solar Heating Systems (Beijing), China Academy of Building Research, Beijing, 100013 China; School of Software, Xiamen University, Xiamen, 361005 China; School of Computing, Informatics, Decision Systems Engineering (CIDSE), Ira A. Fulton Schools of Engineering, Arizona State University, Tempe, AZ 85281 USA

**Keywords:** Water-in-glass evacuated tube solar water heaters, Portable test instruments, Heat collection rate, Heat loss coefficient, Extreme learning machine

## Abstract

**Background:**

Heat collection rate and heat loss coefficient are crucial indicators for the evaluation of in service water-in-glass evacuated tube solar water heaters. However, the direct determination requires complex detection devices and a series of standard experiments, wasting too much time and manpower.

**Findings:**

To address this problem, we previously used artificial neural networks and support vector machine to develop precise knowledge-based models for predicting the heat collection rates and heat loss coefficients of water-in-glass evacuated tube solar water heaters, setting the properties measured by “portable test instruments” as the independent variables. A robust software for determination was also developed. However, in previous results, the prediction accuracy of heat loss coefficients can still be improved compared to those of heat collection rates. Also, in practical applications, even a small reduction in root mean square errors (RMSEs) can sometimes significantly improve the evaluation and business processes.

**Conclusions:**

As a further study, in this short report, we show that using a novel and fast machine learning algorithm—extreme learning machine can generate better predicted results for heat loss coefficient, which reduces the average RMSEs to 0.67 in testing.

## Background

Solar water heaters (SWHs) are powerful and popular techniques to make use of solar energy, which typically use solar collectors and concentrators to gather, store, and use solar radiation to heat air or water in domestic, commercial, or industrial plants (Mekhile et al. 2011). As one of the most important types of stationary collector, evacuated tube solar collectors have substantially lower heat loss coefficient and cost than standard flat plate collectors (Kalogirou 2003; Morrison et al. 1984). In Chinese area, all-glass evacuated tubular solar water heaters are widely used due to their excellent thermal performance, convenient installation, and easy transportability (Shah and Furbo 2007; Liu et al. 2013). In recent years, there are many research groups that focus on the theoretical and experimental studies of the thermal performance of water-in-glass evacuated tube solar water heaters (Pei et al. 2012; Lin et al. 2012; Çomaklı et al. 2012; Govind et al. 2009).

**Fig. 1:**
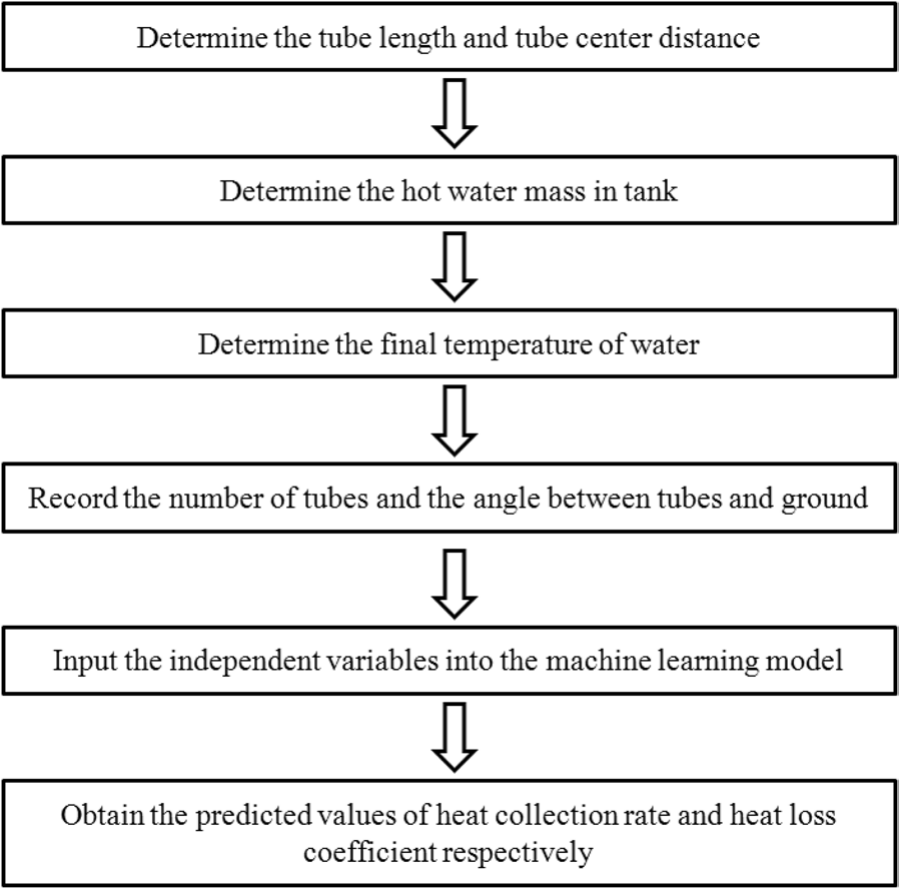
Flow chart of the novel method using “portable test instruments” combined with machine learning models for determining heat collection rate and heat loss coefficient (Liu et al. 2015a)

However, even though we have a testing standard in China (GB/T 4271-2007, Test methods for the thermal performance of solar collectors), there is still few references that show the improved measurements of heat collection rate and heat loss coefficient for solar water heaters, which is a crucial problem for scientists and technicians when evaluating the in service solar water heaters. To solve this problem, we first used “portable test instruments” (Liu et al. 2015a, b), which includes digital thermoelectric thermometer, electric platform scale and taper ZSH-3, to measure the basic properties of water-in-glass evacuated tube solar water heaters. Based on the 915 data groups, artificial neural networks (ANNs) and support vector machine (SVM) were successfully proved to be efficient and precise for predicting the heat collection rates and heat loss coefficients in testing set (Liu et al. 2015a). Compared to conventional measurements, knowledge-based machine learning method is much faster and convenient, saving time, resources and manpower (Liu et al. 2015a). The flow chart of the novel measurement is shown in Fig. 1. To provide a more user-friendly method, the *WaterHeater*, a software based on back-propagation (BP) algorithm in both personal computer (PC) and Android platforms were developed (Liu et al. 2015b). However, in spite of these progresses, there still remains a question that needed to be solved: given that the lowest average RMSEs for the prediction of heat loss coefficient (0.73 in SVM, 0.71 in ANN) is still relatively higher than those of heat collection rates (0.29 in SVM, 0.14 in ANN), can we further improve the RMSEs when predicting the heat loss coefficients? Although the RMSEs of predicting heat loss coefficients are relatively low, which is acceptable to further applications, results show that the precision in predicting the heat loss coefficients can still be improved because their RMSEs in testing is still higher than those of predicted heat collection rates (Liu et al. 2015a, b). Also, in practical applications, even a slight reduction in RMSEs will be considered as significant improvements. To further solve this problem, in this short report, we show that ELM has a lower RMSE for predicting the heat loss coefficients of water-in-glass evacuated tube solar water heaters. Here, ELMs were trained by setting the properties measured by “portable test instruments” as the independent variables. 915 data groups acquired experimentally were used for model training and testing. Comparisons were made between ELMs and our previous models.

## Experimental

### Measurement

In this research, 915 water-in-glass evacuated tube solar water heaters (in service for 1 year) were determined by the “portable test instruments” and the PDT2013-1 (China Academy of Building Research, Beijing, China) detection device developed by the national center for quality supervision and testing of solar heating systems (Liu et al. 2015a, b). Forty-eight PDT2013-1 detection devices were employed to measure the heat collection rate and heat loss coefficient simultaneously. The measured extrinsic properties using “portable test instruments” include tube length, number of tubes, tube center distance, hot water mass in tank, collector area, final temperature and angle between tubes and ground. Table 1 shows the statistical results of the experimental data, which has been reported in our previous work (Liu et al. 2015a, b).

**Table 1:** Statistic of the variables for 915 samples of in service water-in-glass evacuated tube solar water heaters (Liu et al. 2015a, b)

Item	Tube length (mm)	Number of tubes	TCD (mm)	Tank volume (kg)	Collector area (m^2^)	Angle (°)	Final temp. (°C)	HCR	HLC
Maximum	2200	64	151	403	8.24	85	62	11.3	13
Minimum	1600	5	60	70	1.27	30	46	6.7	8
Range	600	59	91	333	6.97	55	16	4.6	5
Average	1811	21	76.2	172	2.69	46	53	8.9	10
SD	87.8	5.8	5.11	47.0	0.73	3.89	2.0	0.48	0.77

*TCD* tube center distance, *temp.* temperature, *HCR* heat collection rate (MJ/m^2^), *HLC* heat loss coefficient [W/(m^3^K)]

### Extreme learning machine (ELM)

ELM is a new single hidden layer feed-forward learning algorithm invented by Huang et al. (2004, 2006) in recent years, which has been proved to have better performances than ANNs and SVM in some scientific cases (Huang 2014). Being similar to the single-layer feed-forward neural network, the network of ELM is a linear system that the input weights and hidden node parameters are selected randomly. The output weights can be obtained by calculating the pseudo-inverse of output matrix of hidden layer. Being different to traditional neural networks, ELM does not need to learn iteratively. The basic advantages of ELM include simple structure, fast learning speed, good global search ability and generalization performance.

For an extreme learning machine with *n* input neurons, *L* hidden layer neurons and *N* training cases trained on a data set $(x_{i} ,t_{i} )$, the mathematical model can be described as:

1 $$\sum\limits_{i = 1}^{L} {\beta_{i} g\left( {a_{i} x_{j} + b_{i} } \right)} = o_{j} ,\quad j = 1, \ldots ,N\quad L \le N$$

where $x_{i} = [x_{i1} ,x_{i2} , \ldots ,x_{in} ]^{T} \in R_{n}$; $t_{i} = [t_{i1} ,t_{i2} , \ldots ,t_{im} ]^{T} \in R^{m}$; $\beta_{i} = \left[ {\beta_{i1} ,\beta_{i2} , \ldots ,\beta_{iL} } \right]^{T}$ is the weight vectors of hidden layer to output layer; $g\left( . \right)$ is the activation of hidden layer neurons; $a_{i} = \left[ {a_{i1} ,a_{i2} , \ldots ,a_{in} } \right]^{T}$ is the weight vectors of input layer to hidden layer; $b_{i}$ is the biases of the neuron in the *i*th hidden layer; and $o_{j}$ is the output value of the *j*th input training sample.

If *L* = *N*, for any *α* and *β*, above model can approximate all the training samples with zero error, namely:

2 $$\sum\limits_{j = 1}^{N} {\left\| {o_{j} - y_{j} } \right\|} = 0$$

thus we have:

3 $$\sum\limits_{i = 1}^{L} {\beta_{i} g\left( {a_{i} x_{j} + b_{i} } \right)} = y_{j} ,\quad \, j = 1, \ldots ,N$$

Which can also be described as:

4 $$H\beta = Y$$

where $Y = \left[ {y_{1}^{T} ,y_{2}^{T} , \ldots ,y_{N}^{T} } \right]_{N \times L}^{T}$; $\beta = \left[ {\beta_{1}^{T} ,\beta_{2}^{T} , \ldots ,\beta_{N}^{T} } \right]_{N \times L}^{T}$; *H* is the output matrix of hidden layer:

5 $$H = \left[ {\begin{array}{*{20}c} {g\left( {a_{1} x_{1} + b_{1} } \right)} & \quad{g\left( {a_{2} x_{1} + b_{2} } \right)} & \quad\cdots & \quad{g\left( {a_{L} x_{1} + b_{L} } \right)} \\ {g\left( {a_{1} x_{2} + b_{1} } \right)} & \quad{g\left( {a_{2} x_{2} + b_{2} } \right)} &\quad \cdots &\quad {g\left( {a_{L} x_{2} + b_{L} } \right)} \\ \vdots & \quad\vdots & \quad\ddots & \quad\vdots \\ {g\left( {a_{1} x_{N} + b_{1} } \right)} & \quad{g\left( {a_{2} x_{N} + b_{2} } \right)} & \quad\cdots &\quad {g\left( {a_{L} x_{N} + b_{L} } \right)} \\ \end{array} } \right]$$

However, when the training sample is large, in order to reduce the amount of calculation, the selection of *L* is usually less than *N*. In this case, the training error of above model approximates an arbitrary value $\varepsilon > 0$, namely:

6 $$\sum\limits_{j = 1}^{N} {\left\| {o_{j} - y_{j} } \right\|} < \varepsilon$$

Therefore, not all parameters of above model need to be adjusted if $g\left( . \right)$ is infinitely differentiable, and parameters *α* and *β* can be selected normally before training and remain unchanged in the training. The weight vectors *β* of hidden layer to output layer can be acquired by solving the following equation set:

7 $$\left\| {H\hat{\beta } - T} \right\| = \mathop {\hbox{min} }\limits_{\beta } \left\| {H\beta - T} \right\|$$

The solution is $\hat{\beta } = H^{ * } \cdot Y$, where $H^{ * }$ is the Moore–Penrose pseudo inverse of matrix *H*. Thus there are only three steps in the training of an ELM:

1. Select the hidden layer neurons *L*.
2. Select an infinitely differentiable function $g\left( . \right)$ as the activation of each hidden layer neuron, and calculate the output matrix *H* (Eq. 5) of hidden layer.
3. Calculate the weight vectors $\hat{\beta }$ ($\hat{\beta } = H^{ * } \cdot Y$) of output layer.

ELM does not need to adjust too many parameters in training, and only needs to adjust the weight $\hat{\beta }$ of hidden layer to output layer by selecting hidden layer neurons *L*. Compared to other existing machine learning techniques, it can acquire the global solution with very short time (Huang 2014). In this study, the data set we used includes 915 data groups, which corresponds to the precondition that fulfills Eq. 6. Therefore, the ELM is considered to be applicable.

## Results and discussion

### Model development

To develop ELM models, we used Matlab software with the package of *basic* ELM (with randomly generated hidden nodes, random neurons) developed by Huang’s research group (ELM code sources). Numbers of hidden nodes were set from 2 to 50 in order to search the best testing results with the lowest RMSEs in testing. Prediction models for heat collection rates and heat loss coefficients were developed respectively. 85 % of data groups were set as the training set. To validate the model, the rest 15 % data groups were set as the testing set, which was to test whether the model was effective in field measurements. Models were trained and tested for 20 times with different components of data groups in training and testing sets, under the same proportions of training and testing sets (85 and 15 %, respectively). The RMSEs were obtained by Eq. 8:

8 $${\text{RMSE}} = \sqrt {\frac{{\sum\nolimits_{i = 1}^{n} {(Z_{i} - O_{i} )^{2} } }}{{n_{tot} }}}$$

where $Z_{i}$ is the predicted value, $O_{i}$ is the actual value and $n_{tot}$ is the number of tested samples.

**Fig. 2:**
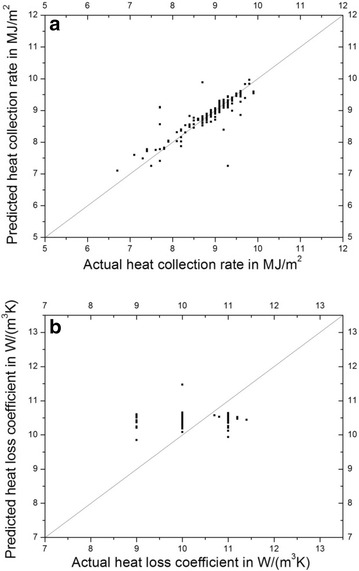
Prediction results for **a** heat collection rates and **b** heat loss coefficients using ELMs

Table 2 shows the selected results of ELMs and previous comparable models (Liu et al. 2015a). The RMSEs shown in Table 2 are the averages of 20 times of training and testing. Results show that the best ELM for heat collection rates exists in the ELM with 31 hidden nodes, with an average RMSE in testing of 0.30. The best ELM for heat loss coefficients exists in 5 hidden nodes, with an average RMSE in testing of 0.67. We can see that the ELM has very good prediction results in both heat collection rates and heat loss coefficients. More importantly, though its average RMSE in predicting heat collection rates is slightly higher than the SVM and the MLFN with 12 nodes (MLFN-12), the average RMSE in predicting heat loss coefficients are lower than previous machine learning methods, which indicates that the ELM can reduce the prediction errors of heat loss coefficients. In practical applications, even this small reduction in RMSE can sometimes significantly influence the evaluation and business of solar water heaters. Therefore, the ELM can be rationally considered as a good alternative machine learning model for predicting heat collection rates and heat loss coefficients for water-in-glass evacuated tube solar water heaters. For further discussions, two representative testing results are shown in Fig. 2, which indicates that the predicted values of heat collection rates using ELM is in good agreement with their actual values (Fig. 2a). Though there is a deviation in the prediction of heat loss coefficient (Fig. 2b) when the actual values are lower or higher than 10 W/(m^3^K), the deviation belongs to the normal data feature of heat loss coefficients because most of the heat loss coefficients of water-in-glass evacuated tube solar water heaters are around 10 W/(m^3^K).

**Table 2:** Prediction results of ELMs and previous machine learning models for heat collection rates and heat loss coefficients

Model	Property predicted	Average RMSE in testing
ELM (31 nodes)	Heat collection rate	0.30
SVM^a^	Heat collection rate	0.29
GRNN^a^	Heat collection rate	0.33
MLFN (12 nodes)^a^	Heat collection rate	0.14
ELM (5 nodes)	Heat loss coefficient	0.67
SVM^a^	Heat loss coefficient	0.73
GRNN^a^	Heat loss coefficient	0.71
MLFN (6 nodes)^a^	Heat loss coefficient	0.73

^a^These results were extracted from Liu et al. (2015a)

## Conclusions

In practical applications of water-in-glass evacuated tube solar water heaters, the reduction of RMSEs, even slight, for predicting heat loss coefficients is a crucial improvement. This is because in practical productions and measurements, technicians usually need to deal with more than thousands of water heaters in a production period, and therefore a slight increase of prediction accuracy rate may help us avoid a decent number of measurement mistakes. To generate improvements for predicting the heat loss coefficients, this short report presents an alternative method for measuring heat collection rates and heat loss coefficients of water-in-glass evacuated tube solar water heaters using ELM. Results show that the ELMs have decent prediction results for heat collection rates and heat loss coefficients, compared to previous study using SVM, GRNN and MLFNs. This study shows that using Matlab software with the package of *basic* ELM can give precise predicted results of heat collection rates and heat loss coefficients with very convenient model development processes. Also, according to the algorithm of the ELM, it can dramatically reduce the required training time (Huang et al. 2004, 2006; Huang 2014), which are usually seen as one of the important advantages for practical applications. In future studies, we’ll focus on developing a completed system with the combination of different machine learning models, taking both RMSEs and required training times into further considerations.

## Abbreviations

ELM: extreme learning machine
ANNs: artificial neural networks
SWH: solar water heaters
RMSE: root mean square error
SVM: support vector machine
TCD: tube center distance
Temp.: temperature (°C)
HCR: heat collection rate (MJ/m^2^)
HLC: heat Loss Coefficient [W/(m^3^K)]
*Z*_*i*_: the predicted value [MJ/m^2^ or W/(m^3^K)]
*O*_*i*_: the actual value [MJ/m^2^ or W/(m^3^K)]
*n*_*tot*_: the number of tested samples (no unit)
MLFN: multilayer feed-forward neural network
GRNN: general regression neural network
